# Osteonecrosis of the jaw induced by receptor activator of nuclear factor-kappa B ligand (Denosumab) - Review

**DOI:** 10.4317/medoral.21044

**Published:** 2016-01-31

**Authors:** Camila-Carvalho de Oliveira, Luiz-André-Cavalcante Brizeno, Fabrício-Bitu de Sousa, Mário-Rogério-Lima Mota, Ana-Paula-Negreiros-Nunes Alves

**Affiliations:** 1 Postgraduate Student in Dentistry (Stomatology and Oral Pathology), Faculty of Pharmacy, Dentistry and Nursing, Federal University of Ceará; 2 Postgraduate Student in Biotecnology, Federal University of Ceará; 3 Associated Professor of Stomatology and Oral Pathology, Department of Dental Clinic, Division of Oral Pathology, Faculty of Pharmacy, Dentistry and Nursing, Federal University of Ceara; 4 Adjunt Professor of Stomatology and Oral Pathology, Department of Dental Clinic, Division of Oral Pathology, Faculty of Pharmacy, Dentistry and Nursing, Federal University of Ceara

## Abstract

**Background:**

Denosumab, an anti-resorptive agent, IgG2 monoclonal antibody for human Receptor activator of nuclear factor-kappa B ligand (RANKL), has been related to the occurrence of osteonecrosis of the jaws. Thus, the aim of this study was to review the literature from clinical case reports, regarding the type of patient and the therapeutic approach used for osteonecrosis of the jaws induced by chronic use of Denosumab.

**Material and Methods:**

For this, a literature review was performed on PubMed, Medline and Cochrane databases, using the keywords “Denosumab” “anti-RANK ligand” and “Osteonecrosis of jaw”. To be included, articles should be a report or a serie of clinical cases, describing patients aged 18 years or over who used denosumab therapy and have received any therapy for ONJ.

**Results:**

Thirteen complete articles were selected for this review, totaling 17 clinical cases. The majority of ONJ cases, patients receiving Denosumab as treatment for osteoporosis and prostate cancer therapy. In most cases, patients affected by ONJ were women aged 60 or over and posterior mandible area was the main site of involvement. Diabetes pre-treatment with bisphosphonates and exodontia were the most often risk factors related to the occurrence of this condition. It is concluded that the highest number of ONJ cases caused by the use of anti-RANKL agents occurred in female patients, aged 60 years or older, under treatment for osteoporosis and cancer metastasis, and the most affected region was the mandible posterior.

**Conclusions:**

The results presented in this article are valid tool supporting the non-invasive mapping of facial vascularization.

**Key words:**Denosumab, osteonecrosis, adverse effects, osteoporosis, antineoplastic protocols.

## Introduction

Denosumab is an IgG2 monoclonal antibody with high affinity and specificity for human Receptor activator of nuclear factor-kappa B ligand (RANKL). It acts as antiresorptive agent, inhibiting osteolysis and blocking interaction between RANKL and RANK (Receptor Activator of Nuclear Factor ᴋ B), preventing osteoclast differentiation and activation (1). This drug has a diferent mechanism of action from bisphosphonates, since it acts on osteoclast precursors, preventing their formation, differentiation and function via inhibition of RANKL action.

In randomized and double-blind studies, Denosumab has proven benefits over bisphosphonates, for providing greater effectiveness and lower acute adverse reactions such as pyrexia and arthralgia, and chronic, including renal toxicity (2). Therefore, Denosumab is recently being used for postmenopausal osteoporosis and prevention of bone metastases.

Although Denosumab presents fewer systemic adverse effects than bisphosphonates and acts by different mechanism of action, evidences indicate that Denosumab is also associated with Osteonecrosis of the Jaws (ONJ) (3). The ONJ is defined as the exposure of necrotic bone in the maxillofacial region for more than eight weeks in patients who a history of exposure to antiresorptive or antiangiogenic agentes and have not been undergone head and neck radiotherapy (4). The fact of the ONJ being associated with both most used antiresorptives agents, strongly suggests that the removal of osteoclasts is critical for the pathophysiology of ONJ. It should be noticed that less potent drugs, such as estrogen and calcitonin, are not associated with this condition, which indicates that the resorption inhibition level affect the development of ONJ (5).

The ONJ is a multifactorial disease, of which occurrence is predisposed by some factors. It includes local factors such as tooth extraction, dent alveolar surgery, periodontal disease, trauma from ill fitting dentures (6) and systemical factors such as malignant diseases (breast, lung and prostate, multiple myeloma), chemotherapy, chronic steroid therapy, smoking, diabetes and anemia (7).

According prospective longitudinal studies phase III, such as Henry *et al.* (3), there was no difference between the incidence of ONJ events found in patients treated for bone metastases with zoledronic acid 4 mg (1.3%) or denosumab 120 mg (1.1%). The same was seen by Stopeck *et al.* (2), in another phase III study, which the incidence of ONJ in patients with breast cancer treated with zoledronic acid (1.4%) was similar to those treated with denosumab (2.0%).

Based on the prospects for therapeutic use in large scale of Denosumab and its possible serious adverse effect on the maxillary bones, the aim of this study was to review the literature from clinical case reports, regarding the type of patient and the therapeutic approach used for osteonecrosis of the jaws induced by Denosumab chronic use.

## Material and Methods

Literature data were carried out on PubMed, Medline and Cochrane databases from January 2010 to May 2015, by using the keywords “Denosumab” or “anti-RANK ligand” AND “Osteonecrosis of jaw”, in both English and Portuguese, together. It was not set any deadline for selection of papers.

Initially, a total of 216 studies were selected based on their titles and abstracts. Subsequently, full-text documents were obtained and a new selection was performed according to the following criteria:

• A case report or a series of clinical cases;

• Patients aged 18 years or over.

• Patients who used denosumab therapy for prevention of bone metastasis and / or treatment of osteoporosis and giant cell tumours;

• Patients who have received any therapy for ONJ.

Only studies in Portuguese, English and Spanish were selected. Clinical trials that compared the effectiveness of Denosumab with other drugs, phase II or III studies, literature reviews, meta-analyses, letters to the editor that does not constitute clinical cases and historical comments were excluded from the study.

Based on the established criteria, 22 articles were selected. These were submitted to a new selection. It was excluded a total of 9 articles: 3 repeated studies, 2 letters to the editor, 2 literature review articles and 2 comparative studies of Denosumab and bisphosphonates, which do not comprise clinical case reports.

An independent review performed the selection. Discrepancies about inclusion or exclusion of studies were solved by extensive discussion between the reviewer and one contributor.

The parameters analysed were: Type of disease treated with anti-RANKL therapy, the characteristics of Denosumab therapy, patient age and sex, site affected by ONJ, local and systemic risk factors, treatment and conclusion of the case report.

## Results

Thirteen complete case reports or case series articles were selected for this review. Some of them constitute a compilation of reports (8-10), totalling 17 clinical cases ( Table 1).

**Table 1:**
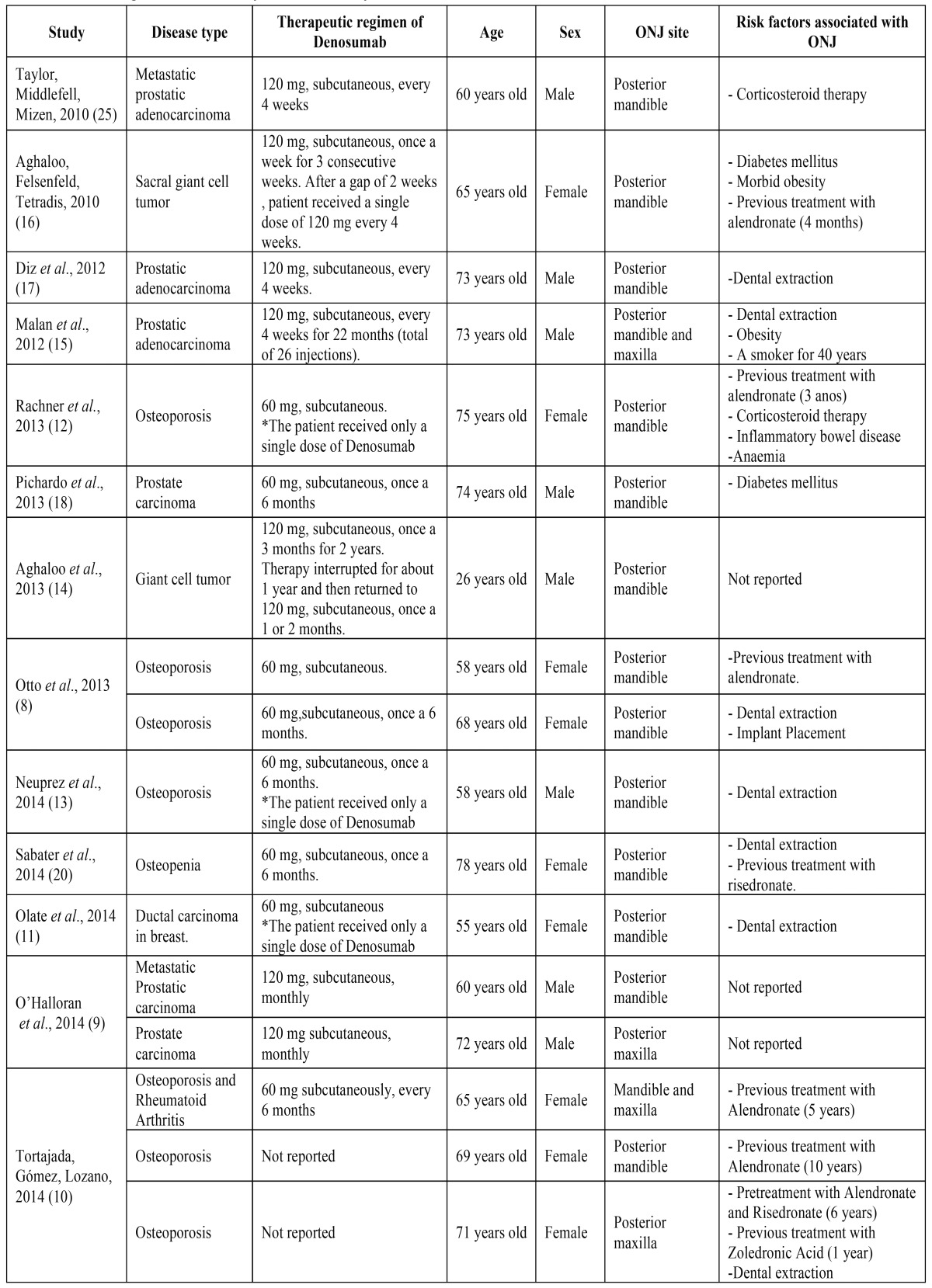
Profile of patients affected by ONJ induced by Denosumab.

The majority of ONJ cases were reported in patients receiving Denosumab as treatment for osteoporosis or osteopenia (47.0%; 8), followed by therapy for prostate cancer (35.3%; 6), giant cell tumour (11.8% 2) and breast carcinoma (5.9%; 1). The therapeutic regimen varied according to aetiology of the disorder treated. In all patients, the drug was administered subcutaneously and doses ranged from 60 to 120 mg of denosumab. Five patients treated for prostate cancer (83.3%) received 120 mg of denosumab every four weeks, for osteoporosis, in general, patients received 60 mg of the drug every six months. Those with giant cell tumour were treated with either three consecutive weekly administrations of 120 mg, continuing with an injection every month or one 120 mg dose of Denosumab every three months, followed by a short-term treatment interruption, then returning to 120 mg, but once a month. Only one patient treated for prostate cancer had a different therapy, receiving 60 mg of the drug every six months ( Table 1).

Three patients, one treated for breast cancer (11) and two others for osteoporosis (12,13), who received a single dose of 60 mg of Denosumab developed ONJ after tooth extraction in posterior mandible area ( Table 1).

In most cases (76.5%; 13), patients affected by ONJ were aged 60 or over; three patients were close to reach that age (58 and 55 years) and another report occurred in a 26-year-old patient (14) ( Table 1).

With regard to sex, nine cases of ONJ were reported in women (53.0%) and eight in men (47.0%) ( Table 1).

The majority (15; 88.2%) of cases of ONJ occurred in posterior mandible area. Two of these patients developed ONJ in posterior maxilla simultaneously (10,15), while two others (11.8%) exhibited the lesion only in posterior maxilla region (9,10) ( Table 1).

The systemic factors associated to the development of ONJ most present were: diabetes, which was reported in the two cases, as well as the treatment with bisphosphonates in seven patients (41.2%). Moreover, an article cited existence of anemia, while others mentioned chronic corticosteroid therapy and smoking. Although hypertension is not directly related to the occurrence of ONJ, it was cited in five (29.4%) reports, while obesity affected two of them (11.8%). Early extraction into the ONJ site was carried out in seven cases (41.2%), acting as a local traumatic factor in predisposing development of the disease ( Table 1).

Systemic and local antibiotic therapy (6; 35.3%) was the most used treatment for ONJ resolution (amoxicillin with or without clavulanic acid associated with mouthwashes with 0.12% chlorhexidine solution). However, clindamycin (1, 5.9%) or penicillin intravenously, especially in patients with trismus, associated with metronidazole (1, 5.9%) were also used. In many cases (7; 41.2%), antibiotic therapy was combined to surgical debridement of necrotic bone exposed. The use of propoxyphene and acetaminophen, non-steroidal anti-inflammatory drugs (NSAIDs), for pain control has been considered in three cases (17.7%). Furthermore, the use of bone anabolic agent (teriparatide) (13) or bone resection fluorescence-guided was performed in one case (5.9%) (8), consisting in other therapeutical options. A case report did not mention the treatment ( Table 2 and  Table 2 Continue).

**Table 2:**
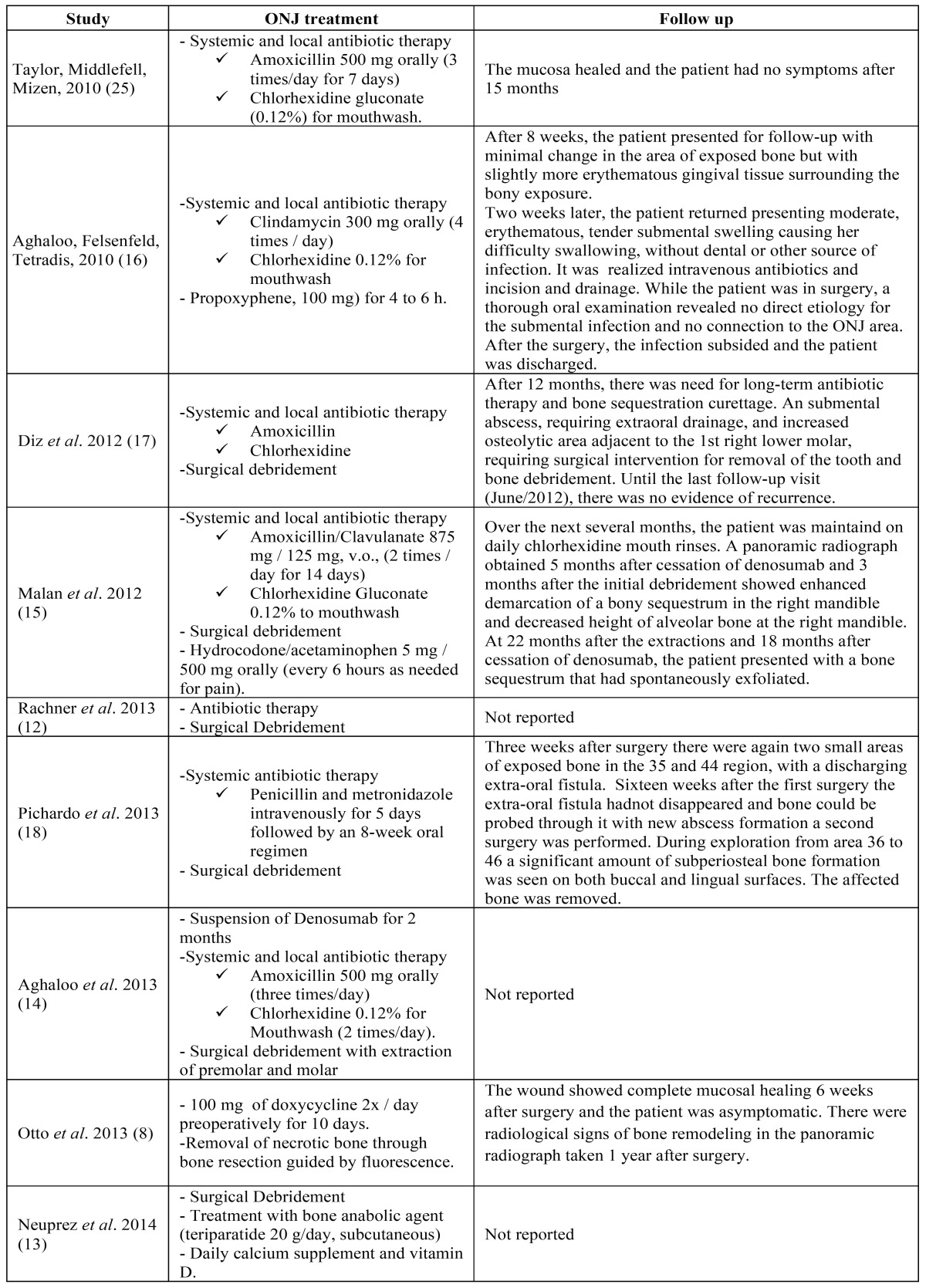
Treatment and follow-up of ONJ induced by Denosumab cases.

**Table 2 Continue:**
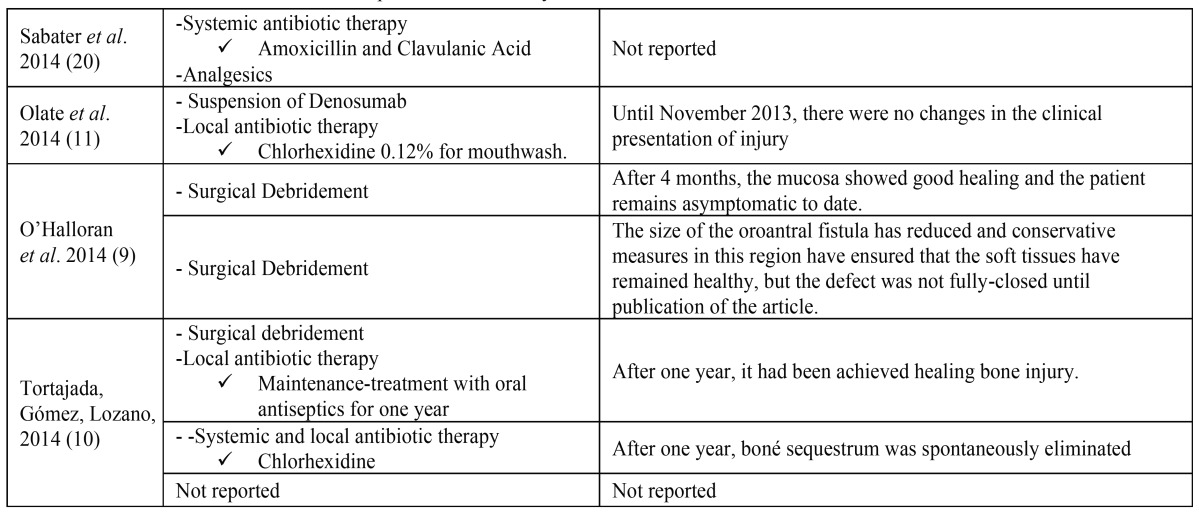
Treatment and follow-up of ONJ induced by Denosumab cases.

After initial therapy, two articles mentioned the need for surgical intervention and three others reported the appearance of abscesses (16-18), two of these were located in submental region (16,17) ( Table 2 and  Table 2 Continue).

## Discussion

The ONJ is a condition closely associated to BPs and its pathogenesis has not been well understood yet (5). However, it is known that this disorder is strongly associated with reduced bone healing caused by antiresorptive drugs. Bone resorption is essential for subsequent neoformation step, which leads to remodelling of this tissue (5). In this literature review, which includes clinical case reports of ONJ patients on therapy with Denosumab published between 2011 and 2014, this relationship was confirmed.

Denosumab is an effective agent in reducing bone loss associated to menopause, markers of bone turnover, reducing new vertebral, hip and non vertebral fractures by 68%, 40%, and 20%, respectively, besides increasing bone mineral density (19). In the present study, most of the clinical cases analysed consisted of Denosumab treatment for osteoporosis and a case described using of this antiresorptive agent in the treatment for osteopenia (20). This drug can still be used effectively in bone metastases therapy, which has great importance, since Denosumab has proved to be very effective in minimizing bone loss associated with breast and prostate cancers (1) and giant cell tumour (21), representing together 53% of cases included in this review.

It is known that according to the type of pathology, there is a particular therapeutic regimen for Denosumab use. For treatment of metastatic bone disease is recommended 120 mg of Denosumab, administered subcutaneously, once every month (2,3), for osteoporosis 60 mg subcutaneously every 6 months (19) and for giant cell tumour treatment, it is required 120 mg every 4 weeks (21). Even though therapeutic regimens are well established, there are cases of patients being treated by following different protocols. This variation may be related to different centers where the treatment is performed. Furthermore, the use of Denosumab is still being researched and, as a consequence, many of the patients were accompanied by phase III studies. However, the accumulated dose and regimen Denosumab employed do not seem to influence ONJ development, since three cases (8-10) reported the occurrence of this event in patients who received only 60 mg of drug. It is known that the risk of ONJ is increased proportionally to the number of bisphosphonates doses, especially when it is combined with potent ones, such as pamidronate and zoledronic acid administered over time (22), differing from ONJ induced by Denosumab, which can occur with fewer infusions of the drug.

The patients who developed ONJ induced by Denosumab are mostly over 60 years old, similar to those patients using BPs (6). Thus, the only young patient who developed this condition was being treated for giant cell tumour (14).

The occurrence of ONJ between the sexes was very similar (9 women; 8 men). However, cases in female patients were directly related to the larger number of case reports in patients with osteoporosis using Denosumab. In male patients, most of the studies demonstrated that Denosumab therapy was used for treatment of prostate carcinoma. Interestingly, there was a case in which a male patient was being treated for osteoporosis (13). The ONJ bisphosphonate-induced is also described in many studies since it affects more females, 67% (7). The preference of that condition in affecting women can be associated with the use of antiresorptive drugs, which is vastly used in treatments for typically female diseases such as osteoporosis and breast cancer. However, some studies point out that there are no gender differences in the development of this condition in patients using bisphosphonates (22).

Locally, some factors may influence the occurrence of ONJ. Among these factors, it is highlighted the presence of periodontitis and the need for extractions (6). Both therapeutic procedures are locally invasive and can be constituted as a source of bacterial infection and inflammation in the periodontal tissues and induce remodelling in the adjacent bone. In such cases, the patient that use antiresorptive agents not have their bone remodelled when submitted to dental intervention, accumulating inflammatory mediators and cells undergoing apoptosis, resulting in an increased risk of developing osteonecrosis (5).

Denosumab has an elimination half-life of approximately 32 days (1). It has been reported that the cessation of osteoclast activity occurs within 6 hours of subcutaneous denosumab injection and returns to normal function approximately 6 months thereafter (9). So, Otto *et al.* (8) recommended that any surgical intervention for ONJ be with held for at least 4 months after denosumab administration to avoid manifestation of ONJ.

The mandible has a greater risk of developing ONJ, since the bone is denser and less vascularized, which further impairs the healing process (23). This risk is even greater in the posterior region, since this site is often subjected to masticatory stimulus, inducing alveolar bone to reshape continuously. Among the studies selected for this review, the majority reported that ONJ was related to the use of systemic Denosumab in posterior mandible area, which confirms results found in the literature. Similarly, the majority of ONJ bisphosphonate-induced also occurs in the mandibular bone, reaching 70% of cases (22).

In addition to local risk factors, it is also known that systemic conditions have been associated with the development of ONJ in patients using bisphosphonates. Diabetics have a less responsive immune system, with lower chemotaxis and activity of polymorph nuclear neutrophils, reduced peripheral vasculature and increased production of inflammatory cytokines, leading the body to a hiperinflammatory state (24), which predisposes a lower response to infections, especially in oral cavity. Furthermore, other factors are also related to ONJ development, such as chronic corticosteroid therapy, anaemia and smoking (6), which can induce changes in the immune response. Among the studies analysed in this review, some patients were diabetic or using corticosteroids, which could contribute to development of ONJ (12,16,18,25).

Although some studies indicate a relation between obesity and development of bisphosphonate-induced ONJ in cancer patients, it is postulated that this event can occur as a consequence of other factors related to cancer treatment. Among these factors, it is highlighted the prolonged use of steroids, which predispose a substantial weight gain and consequent development of obesity. In addition, it is unclear whether obesity-associated ONJ is related to increase masticatory function, which can lead to micro-traumas in previously committed bone tissues. Additionally, obesity increases systemic inflammatory reactions (26), leading to local oxidative stress and it may contribute to the development of bone necrosis. In this literature review, two of the seventeen patients studied were obese (15,16), which does not denote a significant casuist but becomes relevant as it highlights the importance of care to this group of patients.

Some studies revised affirmed that patients who develop ONJ lesions were hypertensive (13,15,16,18). In case-control studies, hypertension was not correlated with increased risk of developing ONJ (7). However, some literature reviews atributtes hypertension as an endogenous risk factor for the ONJ development (27). Another case-report of this review showed that ONJ lesion appeared in a patient with hypothireoidism (16). A large retrospective study affirmed that this comorbidity may be a risk factor for developing ONJ (7). The authors associate hypothyroidism with delayed wound healing, which was seen in previous experimental studies. However, this is the only study to show this relationship. Therefore, a relationship with these comorbities is not well established in the literature and more studies be necessary to elucid these questions.

The therapy for ONJ has not been well established yet. In general, the primary management is done in order to relieve pain, reduce lesion size and minimize inflammation of hard and soft tissue, and/or infection. In most patients who develop ONJ by using bisphosphonates the initial approach is non-surgical and includes antibiotics (such as penicillin, clindamycin, cephalexin and amoxicillin-clavulanate), antifungals (such as fluconazole and nystatin) and mouth rinses containing an antimicrobial solution (chlorhexidine digluconate) (7).

Seven publications (77.7%) opted for treatment with systemic antibiotics associated with mouthwash with chlorhexidine, whereas five of them (55.5%) associated this treatment with surgical/local debridement. Antibiotics were used in order to reduce the infection of exposed bone injury. Some studies show that antibiotic therapy alone is ineffective, making it necessary surgery to remove necrotic bone (16,18). Two (18.2%) clinical cases reported the need for surgical intervention, due to the emergence of a new area of necrotic bone (16,18). The surgical approach is based on the premise that bone exposure, especially with sharp or jagged edges, and the formation of bone sequestrum increase the risk of new infections and inflammation, therefore should be eliminated (28).

In this respect, the bone resection guided by fluorescence can bring many benefits. This treatment involves drug administration (doxycycline) to the pre-surgical patient. This antibiotic, like other tetracyclines, has the ability to be deposited in living bone. Thus, when ingested, this drug reaches the vital bone through the blood stream and produces bright golden-yellow pigment (29) visualized by fluorescence. In this display system (VELscope®), the blue light is emitted (400-460 nm) and the viable bone embedded with doxycycline shows the golden-yellow fluorescence. Since the necrotic bone, which received doxycycline deposits appears in blue colour. Thus, the bone resection is performed until all the necrotic bone is removed, remaining only fluorescent bone, which make the amount of bone to be debrided more precise (8).

Another alternative treatment for ONJ showed in presented case reports reviwed in this study was Teriparatide, a recombinant human N-terminal fragment of parathyroid hormone, is a bone anabolic agent shown to increase bone mass and strength and reduces the incidence of vertebral and non-vertebral fractures in post-menopausal women with osteoporosis (30). In ONJ lesions, teriparatide administration active living bone turnover, causing progression of the separation of the sequestrum followed by normal mucosal coverage of the exposed bone (30) and can show a great effect when this kind of treatment is associated to surgical debridment. However, a case series with ONJ patients in stage 3 lesions showed less favorable results regarding the use of this agent (30). A related disadvantage of teriparatide use is that this drug is contraindicated in patients with metastatic cancer because of their participation in promoting metastasis (30). Despite the controversial results, the study Neuprez *et al.* (13) showed that medication, combined with the suspension of denosumab, may provide fast relief in denosumab-induced ONJ, Although further investigations are needed to better define the role of teriparatide in the treatment of ONJ. The authors affirmed that the increase in bone turnover that occurs when the drug is removed might have a synergistic effect to teriparatide to stimulate bone remodeling and help heal ONJ lesions (13).

In specific cases, it is recommended the use of other drugs, such as clindamycin, an amoxicillin substitute for penicillin-allergic patients (4). Besides, propoxyphene and acetaminophen, both non-steroidal anti-inflammatory drugs (NSAIDs) used to control acute pain, which is a feature of ONJ, as well as intravenous administration of penicillin, particularly performed in patients with trismus, can be used. The suspension of Denosumab after the occurrence and treatment of ONJ has shown a preventive measure to avoid exacerbation of initial condition and, in part, to obtain reversal of bone without remodelling activity (8) but not to reverse ONJ already installed. Unlike bisphosphonates, denosumab is not incorporated into the bone and there is no evidence of drug reciclyng. Therefore, the effects of denosumab on bone turnorver are rapidly reversible with the suspension, and the effect on bone remodeling seems to decrease within 6 months after cessation of treatment (8). In order to prevent ONJ, it is important to maintain a good oral hygiene associated with regular dental visits (6).

## Conclusions

Based on these findings, it is concluded that the highest number of ONJ cases associated with the use of anti-RANKL agents was related to treatment for osteoporosis and metastasis of prostate cancer. This condition was more frequent in female patients aged 60 years or older, the most affected region was the mandible posterior area. Neither the treatment regimen employed nor the duration of Denosumab therapy seems to influence the development of ONJ. However, local factors seem to be important for triggering this adverse effect. Although there is no consensus on the therapy used to treat ONJ yet, the use of mouthwash containing antiseptic solutions and antibiotic therapy associated or not with curettage of necrotic bone has been shown to be an effective treatment in most cases. Nevertheless, it is important further studies to understand the pathogenesis of Denosumab-related ONJ, since anti-RANKL agents have been widely used for therapeutic osteolytic diseases and it was not possible to estimate its prevalence yet.
